# Supplementary material for: Learning curve and surgical outcome of robotic assisted colorectal surgery with ERAS program
Source: Sci Rep. 2022 Nov 29;12:20566. doi: 10.1038/s41598-022-24665-w (PMC9709162; doi:10.1038/s41598-022-24665-w)
Supplement: Supplementary file 1 — Supplementary Information 1. [file 41598_2022_24665_MOESM1_ESM.zip › LSC Raw data-Ñ[▒K/114/O_ñw╝╨░O▒Kñσ.pdf]

正式摘要(電子簽章：000000208648892)

請選擇...reporter

臺中榮民總醫院

Operation Note

[redacted]

- 手術日期：2021/01/21
- 手術醫師：蔣鋒帆，CR 林陳彥，Fellow 江世偉，CR 陳映菁，R 陳凱祥，R 黃瑋
- Anesthesia：GE
- Position：Lithotomy
- Aseptic agents：Betadine Aqua，Zephnan

Diagnosis

- Pre-operative diagnosis : Malignant tumor of sigmoid colon, 7CM IN SIZ, SP ATH, TRY LAP
- Post-operative diagnosis : Locally advanced s-colon tumor with nearly total obstruction and possible para-aortic lymphnode metastasis ? s/p conventional anterior resection (Laparoscopy convert to conventional)

Surgical Indication

- For removing the primary tumor and relieving the obstruction symptoms

Operation

- 1. Conventional, left anterior resection. (Initially tried laparoscopic)
  2. Intraoperative colonoscopy : (-)
  3. IMA high ligation (+). Left colic vessels were also ligated. (Middle colic vessels were not approached.)
  4. EndoGIA (60-4.1) X1 + EEA (33mm) were applied for end to end anastmosis
  5. The colon was taken down from the splenic flexure site and resected
  6. Proximal cut end : > 10 cm, distal cut end : 7 cm
  7. One drainage tube (12 Fr. penrose) was placed over the pelvic (Anastomosis site) area from the LLQ of abdomen
  8. ADHESION BARRIER FILM was placed below the central abdominal wound.

Finding

- 1. A huge tumor at S-colon, which invaded to the nearby abdominal wall and the retroperitonuem area. Multiple enlarged lymphnodes were noted over IMA root and suspicious lymphnodes were also presented at para-aortic region. cT4bN2M1.
  2. No obvious ascites was found.
  3. The left retroperitoneum area was also invaded by the tumor and it was partially resected for adequate safe margin.
  4. Bilateral doughnuts : intact ; Air leak test: Done and no air leak was found

Procedure

- 1. General endotracheal anesthesia was induced and then the patient was placed in the modified Trendelenburg lithotomy position. The abdomen was prepped and draped in standard sterile fashion.

  2. Initially, laparoscopic approach was attempted. Due to locally advanced tumor conditio with suspicious LNs over IMA and para-aortic area, we shfited our procedure to conventional method.
  3. A upper vertical midline incision was made. This was deepened through the subcutaneous tissues and hemostasis was achieved with electrocautery.
  4. The abdomen was explored and wound retractor (Alexis) was set up. Operative findings were as mentioned above.
  5. The small bowel was inspected and pulled out using a moist pad.
  6. The peritoneum overlying the mesentery was then scored with electrocautery. Multiple hardened

- mass lesion, in favor of LNs were found along the IMA region. The IMA root was ligated with Harmonic scalpel and with silks. The left colic artery was also identified, ligated with silks, then transected. IMV was also ligated with 2-O silk. (The para-aortic lymphnodes were resected for sampling as well.)
  7. The lesser sac was approached and the omentum was resected carefully. The gastrocolic omentum was divided as far to the left as possible until the splenic flexure was no longer tethered. The left branch of the middle colic artery was also indentified and remained intact.
  8. The avascular line of Toldt along the left paracolic gutter was incised from the sigmoid colon to splenic flexure as possible. The retroperitoneum tissue was partially dissected due to tumor invasion.
  9. The descending colon was partially lifted and rotated medially. Incising the additional lateral peritoneal coverings further mobilized the colon. The distal part of the colon (R-S junction) was resected via EndoGIA (60-4.1) first for better operative field and easier mobilization.
  10. The tumor part was severely attached to the gerota fascia, which was divided carefully via the LIGASURE. Hemostasis was checked in the operative field. The bilateral ureter were all identified and protected carefully.
  11. After the descending colon and sigmoid colon were nearly fully mobilized, the point for proximal transection was selected. (Distal T-colon)
  12. A pursestring suture of 2-0 prolene was placed. The anvil was inserted, and then the pursestring suture was tied. (The specimen was also removed smoothly)
  13. The anal canal was irrigated with distil water and then the circular stapler (EEA 33 mm) was introduced through the anus and pierced through the mid-portion of the stapler line.
  14. The proximal bowel with anvil in place was engaged with the circular stapler and the stapler was then fired. Two complete donuts were obtained and the distal one was submitted to pathology for margin confirmation.
  15. The anastomosis was tested for leaks with irrigation solution and air introduced through the rectum and was found to be patent and intact.
  16. Possible bleeder was checked again thoroughly.
  17. A 12 Fr. penrose drain was placed at pelvic site from LLQ of the abdomen.
  18. The fascia was closed with interrupted suture via Num.1 Vicryl.
  19. After the wound was fully irrigated, the skin was closed with interrupted mattress suture via 3-O Nylon, then covered with gauze.
  20. The NG tube was then removed.
  21. The patient tolerated the procedure well and was brought to the POR under stable condition.
- Estimated blood loss: minimum
- 器械數量: 正確
- 紗布數量: 正確

Image

- PAINT
  - abdomen

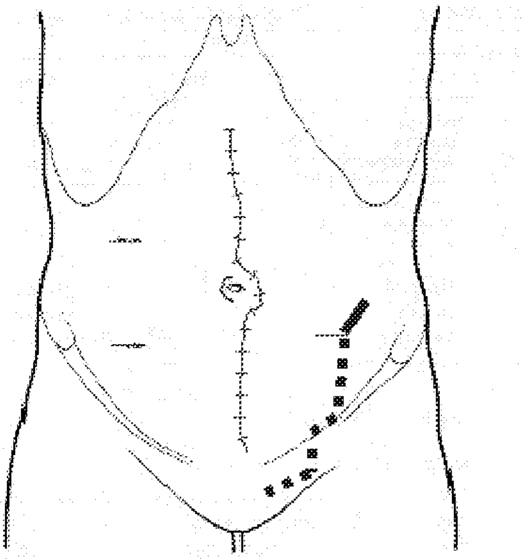

- colon & rectum

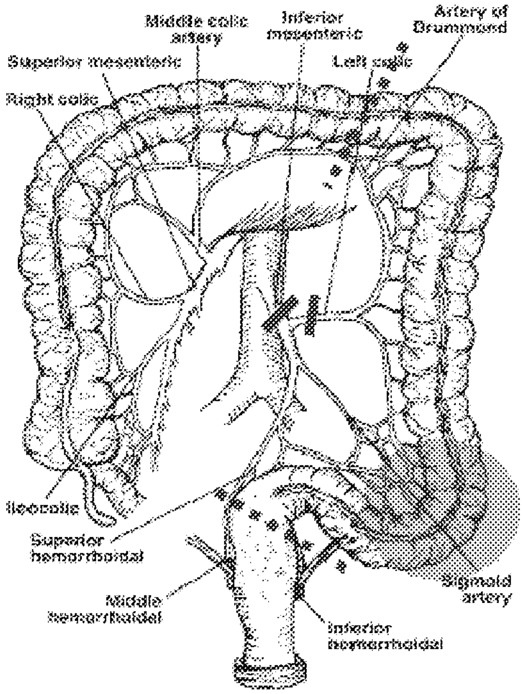

- The resected specimen

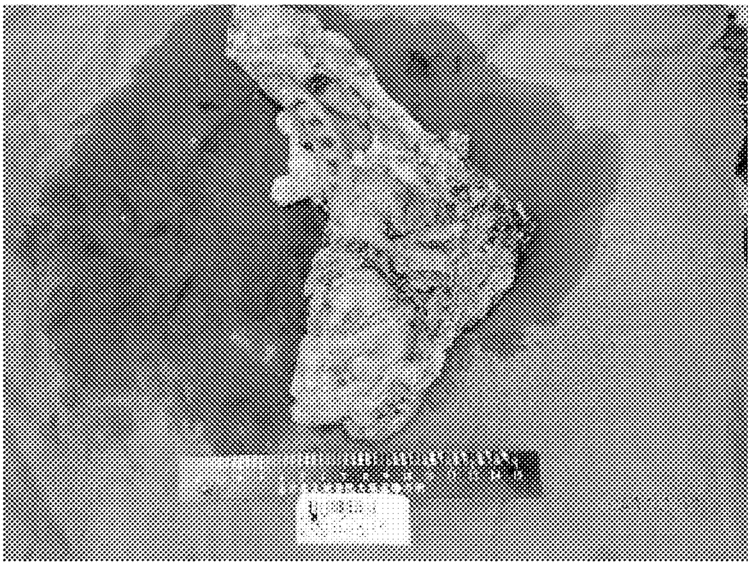

The nearly total obstruction tumor

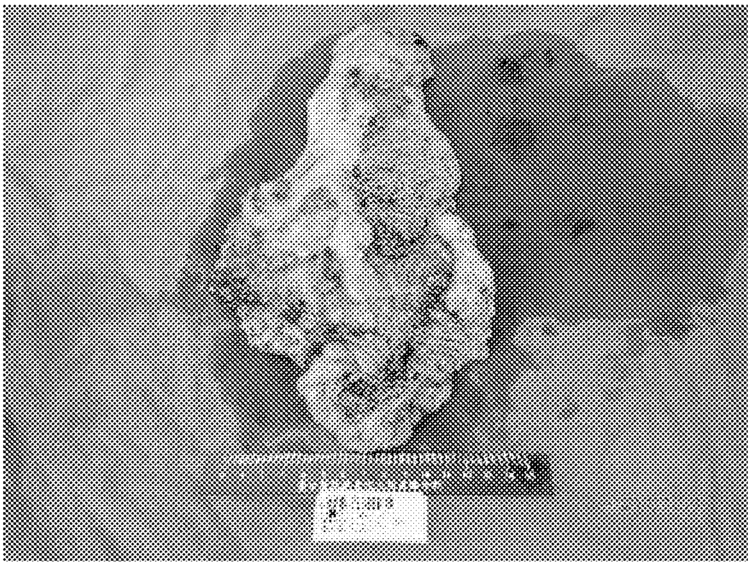

Multiple enlarged lymphnodes along the IMA root were also presented

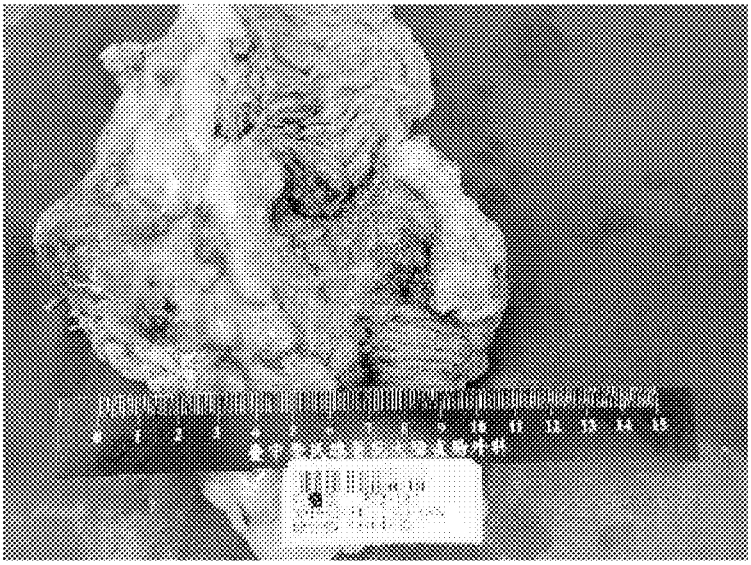

主任 2062F蔣鋒帆 / 醫師 2301H林陳彥

正式摘要(電子簽章：**000000208648892)**

請選擇...reporter

doctorRank:B
